# Moral elevation and prosocial behavior in college students: the mediating role of gratitude and the moderating role of empathy

**DOI:** 10.3389/fpsyg.2026.1784102

**Published:** 2026-03-24

**Authors:** Yuemei Zhang, Mingwei Bu, Haitao Liu

**Affiliations:** 1 School of Marxism, Jiamusi University, Jiamusi, Heilongjiang, China; 2 College of Educational Science, Jiamusi University, Jiamusi, Heilongjiang, China

**Keywords:** empathy, gratitude, moderating effect, moral elevation, prosocial behavior

## Abstract

**Introduction:**

With growing attention to moral development in society, the moral cognition and behavior of college students have become important topics of interest. Moral elevation may influence individuals’ sense of obligation toward others and is thought to relate to prosocial conduct through affective pathways, including but not limited to gratitude. Grounded in the Social Intuitionist Model and the Empathy–Altruism Hypothesis, this study proposes a moderated mediation model to explore the relationship between moral elevation and prosocial behavior, with gratitude as a mediator and empathy as a moderator.

**Methods:**

Using standardized questionnaires, data were collected from 1,261 college students on moral elevation, gratitude, empathy, and prosocial behavior. Among the participants, 54.08% were male (*n* = 682), and the majority were between 18 and 22 years old (97.46%). This study used the Bootstrap method with SPSS PROCESS macro 4.2 to test the model.

**Results:**

The results suggest that moral elevation is positively associated with prosocial behavior, and that gratitude may play a mediating role in this association. Furthermore, empathy appears to moderate the relationship between moral elevation and prosocial behavior, with the effect being more pronounced among individuals with lower levels of empathy.

**Discussion:**

This study explores the role of gratitude and empathy in connecting moral elevation with prosocial behavior in college students. These findings yield important implications for theory development and practical applications within educational and psychological domains, particularly in promoting gratitude, enhancing empathy, and fostering prosocial behavior.

## 1 Introduction

Prosocial behavior refers to a broad range of actions considered beneficial to others by key social groups and/or members within a society (Penner et al., 2005). Engaging in academic and social experiences at the university level provides young adults with a foundation for constructing belief systems and cultivating the skills needed to participate effectively in social contexts. During this stage, prosocial behavior may facilitate healthy interpersonal relationships and psychological adjustment and reflect the internalization of social responsibility and moral awareness (Ferguson, 2015; Jin et al., 2024; Zhou, 2025). Contemporary research primarily explores the influencing mechanisms of prosocial behavior from three dimensions: formative mechanisms, processing mechanisms, and boundary mechanisms. Formative mechanisms emphasize how relatively stable factors—such as neural substrates, personal values, and personality traits—shape prosocial tendencies across development (Paulus, 2014; Wu and Hong, 2022). Processing mechanisms focus on how emotional and cognitive processes are activated and translated into prosocial behavior within specific situational contexts (Decety et al., 2016). Boundary mechanisms examine the conditions under which the above effects may be strengthened or weakened due to individual differences or contextual moderators (Wu et al., 2022). Among college students, existing studies have mainly focused on processing and boundary mechanisms, providing empirical insights into their social functioning and offering evidence-based implications for moral education in higher education.

Moral elevation, defined as a moral emotion triggered by observing virtuous acts performed by others, has been identified as a key antecedent of prosocial conduct among college students (Aquino et al., 2011; Haidt, 2003b). Gratitude, as a positive emotional response to others’ goodwill or benevolence, is regarded as a vital emotional bridge linking moral cognition to behavioral practice, and it may also foster prosocial behavior (McCullough et al., 2001). Moreover, individual traits may influence the extent to which moral elevation promotes prosocial action. Key factors, including personal traits like empathy and agreeableness, the capacity for emotional self-regulation, and moral development, all play crucial roles in whether moral emotions lead to concrete actions aimed at helping others (Batson et al., 1997; Eisenberg and Lennon, 1983; Martin-Raugh et al., 2016). Specifically, empathy, as an essential interpersonal competency, facilitates understanding and emotional alignment with others, thereby promoting various forms of prosocial engagement (Preston and de Waal, 2002). However, the pathway linking moral elevation, gratitude, and prosocial behavior remains underexplored, especially with respect to integrated models that simultaneously incorporate both mediating and moderating mechanisms. Therefore, grounded in Social Intuitionist Model (Haidt, 2001) and the Empathy–Altruism Hypothesis (Batson, 1991), this study proposes a moderated mediation model in which gratitude serves as a mediator and empathy functions as a moderator, aiming to uncover the specific psychological mechanisms through which moral emotions are transformed into prosocial actions among college students. The purpose of this research is to enrich conceptual insights into prosocial tendencies while providing empirical support for improving moral development programs targeted at college students.

## 2 Literature review

### 2.1 Theoretical foundation

To enhance the scientific rigor and theoretical persuasiveness of the proposed model, the present study integrates Social Intuitionist Model and the Empathy–Altruism Hypothesis, and develops a hypothesized framework on this basis.

First, the Social Intuitionist Model (Haidt, 2001) posits that moral judgments and subsequent behaviors are primarily driven by quick, affectively laden moral intuitions rather than slow, conscious reasoning. Within this framework, moral emotions serve as the proximal motivators that translate intuitive evaluations into action (Haidt, 2003b). Among these, gratitude is regarded as an important moral emotion that typically arises from affective responses to others’ virtuous actions, which can stimulate motivations of reciprocity, moral obligation, and prosocial engagement (McCullough et al., 2001). Although moral elevation and gratitude have been previously linked, the theoretical connection between them remains under-explored. Gratitude has been shown to enhance prosocial behaviors (Sheng and Fang, 2024), and moral elevation may play a role in eliciting feelings of gratitude, further strengthening prosocial actions (Oguni and Ishii, 2024). The Social Functional Theory of Emotions emphasizes that gratitude helps reinforce social bonds and fosters altruism in interpersonal interactions (Algoe, 2012). Therefore, grounded in the Social Intuitionist Model’s emphasis on affective drivers, the present study posits that gratitude serves as a mediator in the connection between moral elevation and prosocial actions, offering this as an exploratory hypothesis.

Second, to further understand the conditional factors that influence whether moral elevation translates into concrete behavioral expressions, the study adopts the Empathy–Altruism Hypothesis as the theoretical foundation for the moderating mechanism. Proposed by Batson (1991), this hypothesis suggests that when individuals experience emotional resonance or empathic concern for others, they are more inclined to act in prosocial ways driven by purely altruistic motivation. In other words, even when gratitude is elicited, varying levels of empathy may lead to different behavioral outcomes (Pang et al., 2022). Thus, empathy may influence how gratitude, once elicited, manifests in actions. Empathy is crucial for recognizing the needs of others and responding to moral cues, ultimately encouraging prosocial actions and a greater sense of care for others (Batson, 1991; Batson, 2010). Unlike transient, event-induced emotions, empathy is often conceptualized as a relatively stable individual difference or ability structure; hence, it is more appropriate to be modeled as a moderator explaining variations in the strength of moral pathways, rather than as a mediating variable (Batson, 1991; Davis, 1983). To understand the varied behavioral effects of gratitude, one must consider empathy as a moderator, specifically regarding one’s dispositional capacity for empathy.

In summary, this study constructs the mediating path based on Social Intuitionist Model and establishes the moderating mechanism under the framework of the Empathy–Altruism Hypothesis, aiming to address existing research gaps while enhancing the theoretical depth and explanatory capacity of the model. The integrated model not only broadens the understanding of the mechanisms underlying moral elevation but also provides a theoretical basis for moral education and social behavior interventions in the academic contexts.

### 2.2 Moral elevation and prosocial behavior

With the rise of positive psychology, Haidt (2003a) began to explore whether the moral emotion he had long studied—moral disgust—might have a positive counterpart. Consequently, the term “moral elevation” was coined to describe an emotionally positive reaction that is often evoked when individuals witness behaviors that reflect high moral standards, altruism, or exemplary ethical conduct (Algoe and Haidt, 2009; Haidt, 2003b). Experiencing moral elevation can generates affective states such as deep admiration, emotional warmth, and an intrinsic motivation to pursue personal moral growth. Importantly, this emotion can arise even when the observer is not the direct beneficiary of the moral act (Thomson and Siegel, 2017).

Among the factors influencing prosocial behavior, moral elevation has been shown to directly promote prosocial behaviors among college students (Algoe and Haidt, 2009). According to Social Intuitionist Model, moral emotions such as moral elevation serve as a bridge between moral cognition and overt behavior by triggering altruistic motivation and prosocial engagement when individuals are exposed to others’ virtuous acts (Tangney et al., 2007). In a set of experimental studies, Thomson and Siegel (2013) demonstrated that observing prosocial behaviors tended to trigger moral elevation, which subsequently increased individuals’ propensity to engage in prosocial actions. The association between moral elevation and prosocial tendencies has been well-documented in prior investigations involving both adolescent and college-aged samples. For instance, Wang et al. (2025) identified moral elevation as a crucial bridge linking moral identity internalization to prosocial behavior, while Ding et al. (2018) found that higher levels of moral elevation among Chinese college students were linked with greater willingness to participate in volunteer activities. Furthermore, Li et al. (2022) conducted a manipulation experiment and discovered that observing punitive consequences following prosocial behavior may suppress moral elevation and reduced prosocial responses, suggesting that the effect of moral elevation on behavior may be contextually sensitive. Such results underscore moral elevation as a potent motivational catalyst for prosocial action, rather than simply a fleeting affective state.

### 2.3 The mediating effect of gratitude

Gratitude encompasses both an emotional reaction to receiving assistance from others and a consistent tendency to value the favorable elements present in one’s daily experiences (Haidt, 2003b; Wood et al., 2010). The Social Intuitionist Model proposes that emotions such as gratitude, shame, guilt, and elevation link moral cognition to moral behavior (Tangney et al., 2007). Unlike general positive emotions, moral emotions are inherently other-oriented, involving concern for social norms and the welfare of others, thereby guiding individuals from emotional experience toward moral action. As a prototypical moral emotion, gratitude can elicit reciprocity and altruistic motivation, which may foster prosocial behavior (McCullough et al., 2004). Gratitude has been shown to enhance individuals’ perceived social support and life satisfaction, protecting them from stress and depression, and playing an important role in clinical interventions for mental health (Wood et al., 2008a,b). Gratitude not only enhances emotional well-being but also acts as a multidimensional ethical mechanism that guides individuals’ awareness of virtuous acts, motivating them to uphold prosocial values, and strengthening the continuity of moral practices across interpersonal contexts (McCullough et al., 2001). Cross-cultural evidence further supports a robust association between gratitude and prosocial behavior among college students (Oguni and Ishii, 2024; Oguni and Otake, 2025; You et al., 2022; Zhu et al., 2024). For example, Oguni and Otake (2025) found that gratitude could activate generous motivations in college students, thereby increasing their prosocial tendencies. Distinguishing subtypes of gratitude has enabled researchers to explore their unique contributions to prosocial behavior. Zhu et al. (2024), drawing on both cross-sectional and longitudinal data, concluded that only general gratitude significantly predicted prosocial tendencies among college students, whereas affective and cognitive gratitude showed no direct associations.

From the perspective of emotional chains, moral elevation is considered an important antecedent of gratitude. Both emotions belong to the class of other-praising emotions, yet they differ in their eliciting conditions: moral elevation typically arises from witnessing others’ virtuous acts, while gratitude is more likely to emerge when individuals personally benefit from others’ benevolence (Algoe and Haidt, 2009; Thomson and Siegel, 2017). Unlike simple thankfulness, moral elevation is thought to deepen individuals’ understanding of the moral significance of their own actions and strengthen their intrinsic moral motivation (Siegel et al., 2014). The experience of moral elevation appears to exert its transformative power by eliciting gratitude, which in turn broadens an individual’s sense of purpose and promotes altruistic action (Pohling and Diessner, 2016; Pohling et al., 2018; Sheng and Fang, 2024). In a cross-sectional survey, Sheng and Fang (2024) found that higher levels of moral elevation are associated with greater gratitude in college populations. Further, studies by Pohling et al. (2018) and Pohling and Diessner (2016) have shown that moral elevation not only evokes feelings of admiration and respect for others’ virtuous actions but also contributes to an increased sense of gratitude, which subsequently fosters prosocial behavior. Taken together, gratitude is considered not merely a positive affective response but also an emotion carrying moral information, serving as a crucial psychological channel that transforms the moral cognition evoked by moral elevation into observable prosocial behavior. Therefore, grounded in Social Intuitionist Model and supported by empirical evidence, this study proposes that gratitude mediates the relationship between moral elevation and prosocial behavior—linking emotional activation with behavioral expression through a morally infused affective pathway.

### 2.4 The moderating effect of empathy

As a socio-emotional construct, empathy is thought to be shaped by both stable personality characteristics and the immediate situational context in which an interaction occurs (Cuff et al., 2016). Contemporary scholars generally conceptualize empathy as comprising both affective and cognitive components (Davis, 2006). On the affective level, empathy refers to an observer’s ability to resonate with or emotionally respond to the emotional states of others (Batson, 1991). This connection allows individuals to emotionally engage with others, potentially leading to stronger incentives to participate in prosocial actions. On the cognitive level, it refers to the skill of recognizing and interpreting others’ internal experiences (Wispé, 1986). Cognitive empathy, therefore, allows individuals to comprehend others’ emotional states from a rational standpoint, which may contribute to more thoughtful and deliberate helping behaviors. Both empathy and the capacity to elicit empathy from others are considered essential to appropriate moral development (Jolliffe and Farrington, 2006). However, these dimensions may have different roles in how individuals process moral emotions and engage in prosocial behaviors. Affective empathy is more directly linked to emotional responses, which can drive immediate helping actions, while cognitive empathy may influence the way individuals interpret and appraise the specific vulnerabilities of others, potentially leading to more calculated, less impulsive prosocial behavior.

According to the Empathy–Altruism Hypothesis (Batson, 1991; Batson, 2010), individuals who experience empathic concern for others’ welfare frequently exhibit helping tendencies that are intrinsically altruistic, as opposed to acting out of instrumental considerations for rewards or penalties. This framework suggests that empathy can strengthen the emotional-to-behavioral transformation pathway. In the present study, moral elevation—as an emotionally induced state—may influence prosocial behavior depending on individuals’ sensitivity to others’ situations and their emotional processing style (Haidt, 2003b). For instance, individuals who exhibit elevated empathy are more inclined to emotionally resonate with others’ situations and may convert moral affect into observable prosocial behaviors. In contrast, those with lower levels of empathy may lack sufficient emotional activation or motivation to act, even when exposed to the same moral stimuli (Ringwald and Wright, 2021). Empirical evidence supports this assumption. Li et al. (2022) found that moral elevation was significantly associated with prosocial behavior only among adolescents with low levels of empathy, whereas this association was non-significant among high-empathy individuals. The findings suggest that empathy levels may shape how people perceive social expectations, evaluate resources, and foresee behavioral outcomes, ultimately moderating how moral elevation impacts their intentions to act (Ge et al., 2023). Affective empathy might particularly strengthen the emotional response to moral elevation, while cognitive empathy might enhance the ability to assess the appropriateness of prosocial behaviors, making it a crucial moderator of the relationship between moral elevation and prosocial behavior relationship. Despite growing interest in empathy and moral emotions, empirical investigations into how individual differences in empathic capacity influence the association between moral elevation and prosocial behavior among college students remain scarce. Exploring this moderation helps clarify how individual traits bridge moral motivation and action, illuminates the boundary conditions of prosociality, and inform theory-based, personalized social–emotional interventions.

### 2.5 The current study

**Figure 1:**
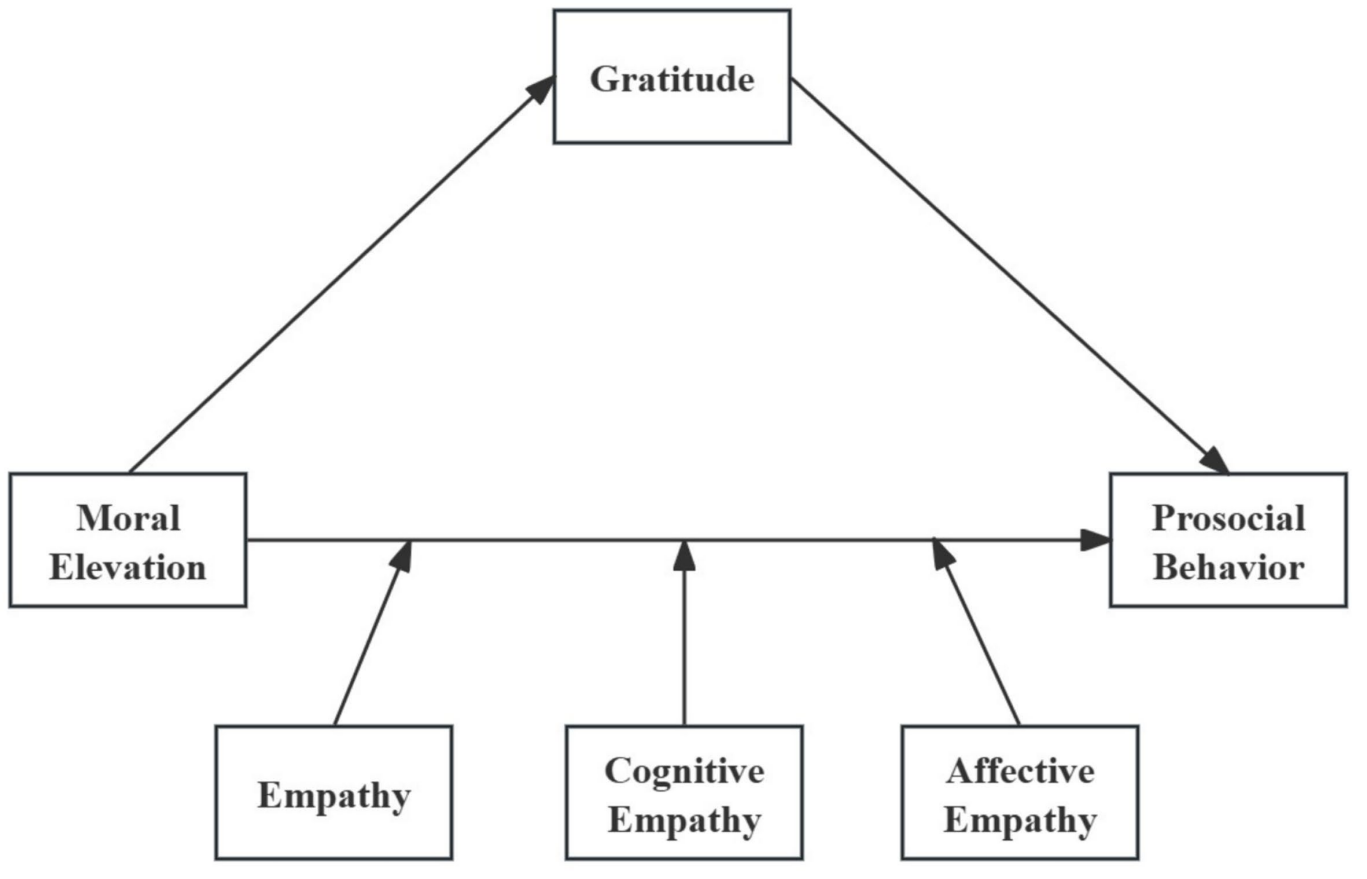
The research model.

Research on moral elevation in higher education is sparse, with insufficient attention given to the mediating or moderating processes that might underlie its influence on prosocial behavior. Grounded in the Social Intuitionist Model and the Empathy–Altruism Hypothesis, the present study addresses the gap by proposing a model in which gratitude mediates and empathy moderates the effect of moral elevation on prosocial behavior. Affective empathy is believed to strengthen the emotional reaction to moral elevation, while cognitive empathy enhances the ability to evaluate whether prosocial behavior aligns with moral norms, with both acting as moderators in this relationship. Research hypotheses and the conceptual framework are provided in Figure 1.

*H1*: Moral elevation is positively associated with prosocial behavior.

*H2*: Gratitude mediates the relationship between moral elevation and prosocial behavior.

*H3a*: Empathy moderates the relationship between moral elevation and prosocial behavior.

*H3b*: Affective empathy moderates the relationship between moral elevation and prosocial behavior.

*H3c*: Cognitive empathy moderates the relationship between moral elevation and prosocial behavior.

## 3 Methods

### 3.1 Participants and procedure

Data were gathered via self-report surveys from students at several universities in Northeast China between May and June 2025. The questionnaire was designed and distributed using the online platform Wenjuanxing. With official clearance granted by the offices of academic affairs and associated departments, the research team proceeded to carry out participant sampling and data collection. Senior (fourth-year) students were excluded from the sampling frame due to their limited availability during data collection, as they were either preparing for graduation, undertaking internships, or engaged in job-seeking activities off campus. These transitional experiences might have substantially altered their emotional state and behavioral patterns, leading to inconsistent responses compared to students in earlier years. This exclusion was implemented to ensure internal consistency and comparability in the data. Considering the large student population, a stratified random sampling method was employed. First, all classes were assigned a numeric code, and 50 classes were randomly selected to form the total sampling pool. Next, students within those 50 classes were re-coded, and 1,500 students were randomly selected as participants.

The research team visited students’ classrooms and invited them to complete the questionnaire during break times by scanning a QR code or clicking a survey link. An informed consent script was read aloud by trained assistants before survey administration, informing participants of the study’s purpose, expected duration (around 10 min), data confidentiality, and their right to voluntary and penalty-free withdrawal at any stage. The questionnaire collected only non-sensitive demographic information relevant to the study (e.g., academic year, major). Students proceeded to the survey platform only after confirming informed consent. This study was approved by the Ethics Committee of Jilin Railway Technology College (Approved No. 2025-0049), and all procedures followed the ethical principles of Declaration of Helsinki.

A total of 1,311 questionnaires were returned. After excluding 26 incomplete responses and 44 responses with poor quality (e.g., excessively short or long completion times, over 80% of responses selecting the same option), 1,261 valid questionnaires remained, yielding a valid response rate of 94.74%.

Demographic characteristics of the participants are presented in Table 1. Among the 1,261 respondents, the gender distribution was relatively balanced, with a slightly higher proportion of male students (*n* = 682, 54.08%). Participants’ ages were reported in four categories: under 18 years (*n* = 14, 1.11%), 18–20 years (*n* = 910, 72.16%), 20–22 years (*n* = 319, 25.30%), and over 22 years (*n* = 18, 1.43%). In total, 97.46% of the sample were between 18 and 22 years, consistent with the conventional age range of Chinese college students. Because age was collected in categories, we report the distribution rather than a mean; the median and modal category is 18–20 years. Most participants were first- or second-year students, while third-year students accounted for only 5.31%. Most participants were freshmen or sophomores, while juniors represented a smaller proportion (5.31%). In terms of academic disciplines, over half of the participants were majoring in social sciences (60.19%), followed by engineering (28.07%), and a smaller proportion were enrolled in natural sciences (11.74%). This distribution largely reflects the academic offerings and accessibility of students at the participating institutions.

**Table 1:** Frequency analysis of demographic variables.

Classification	Frequency	Percentage
Gender		
Male	682	54.08%
Female	579	45.92%
Age		
Under 18 years old	14	1.11%
18–20 years old	910	72.16%
20–22 years old	319	25.30%
Over 22 years old	18	1.43%
Grades		
First year	759	60.19%
Second year	435	34.50%
Third year	67	5.31%
Major		
Humanities	354	28.07%
Social sciences	759	60.19%
Natural sciences	148	11.74%

### 3.2 Measurements

The study employed two types of measures: demographic items and validated psychological scales targeting the key variables. For linguistic appropriateness, all English instruments were translated into Chinese through a rigorous back-translation process (Brislin, 1980) conducted by bilingual professionals.

Moral elevation was measured using the Chinese version of the Moral Elevation Scale developed by Ding et al. (2014). This instrument has demonstrated strong reliability and validity in previous research with Chinese samples (Ding et al., 2018; Fang and Huang, 2023). The scale contains 21 items rated on a 5-point Likert scale and comprises four dimensions: emotional experience and expression (8 items), self-perception (4 items), behavioral tendency (5 items), and perception of others (4 items). In the current study, the scale demonstrated excellent internal consistency (Cronbach’s *α* = 0.950) and high sampling adequacy (KMO = 0.979), with Bartlett’s test of sphericity being significant (*p* < 0.001). McDonald’s Omega for this scale was also 0.950. Confirmatory factor analysis (CFA) indicated good model fit: *χ*^2^/df = 2.817, RMSEA = 0.038, GFI = 0.962, AGFI = 0.951, IFI = 0.977, CFI = 0.977, and TLI = 0.973.

Prosocial behavior was assessed using the Prosocialness Scale for Adults (PSA) developed by Caprara et al. (2005). The Chinese version has been validated in prior studies (Lui et al., 2022; Zhan et al., 2023). This unidimensional scale consists of 16 items rated on a 5-point Likert scale. In the present sample, the PSA exhibited high internal consistency (Cronbach’s *α* = 0.943) and strong sampling adequacy (KMO = 0.971), with Bartlett’s test of sphericity reaching significance (*p* < 0.001). Additionally, McDonald’s Omega for the PSA was 0.944. CFA results supported the measurement model: *χ*^2^/df = 4.977, RMSEA = 0.056, GFI = 0.949, AGFI = 0.930, IFI = 0.967, CFI = 0.967, and TLI = 0.960.

Gratitude was assessed using the Gratitude Questionnaire-6 (GQ-6) developed by McCullough et al. (2002). Prior research has confirmed the reliability and validity of the Chinese adaptation in studies with Chinese participants (Chan, 2010; Guo et al., 2023). The instrument comprises six items scored on a 7-point Likert scale, with items 3 and 6 reverse-coded, and functions as a unidimensional measure. In the present sample, the GQ-6 demonstrated strong internal consistency (Cronbach’s *α* = 0.925), a KMO value of 0.914, and a significant Bartlett’s test of sphericity (*p* < 0.001). McDonald’s Omega for this scale was 0.927. CFA indicated satisfactory model fit: *χ*^2^/df = 5.997, RMSEA = 0.063, GFI = 0.992, AGFI = 0.966, IFI = 0.995, CFI = 0.995, and TLI = 0.986.

Empathy was measured using the Basic Empathy Scale (BES) developed by Jolliffe and Farrington (2006). The Chinese version has been validated in earlier work (Geng et al., 2012) and more recent studies (Wu et al., 2022), demonstrating robust psychometric properties. The BES comprises 20 items rated on a 5-point Likert scale, with items 1, 6, 7, 8, 13, 18, 19, and 20 reverse-scored. It contains two subscales: cognitive empathy (9 items) and affective empathy (11 items). In the current sample, the BES demonstrated excellent internal consistency (Cronbach’s *α* = 0.948) and strong sampling adequacy (KMO = 0.960), with Bartlett’s test of sphericity being significant (*p* < 0.001). McDonald’s Omega for this scale was 0.948. Although the error values in the CFA were somewhat high and the fit values were moderate, the results still suggest acceptable model fit: *χ*^2^/df = 8.750, RMSEA = 0.078, GFI = 0.904, AGFI = 0.845, IFI = 0.937, CFI = 0.937, and TLI = 0.909. Despite the moderate fit indices, the BES has shown adequate validity and reliability for use in the Chinese population.

### 3.3 Statistical analysis

The analysis strategy involved descriptive statistics, correlation testing, assessment of common method bias, model fit evaluation, and conditional process analysis. Descriptive and correlation analyses were conducted using SPSS 26.0. Potential method bias was checked prior to model testing. AMOS 26.0 was used for structural validation. Specifically, Model 4 was initially applied to confirm the mediating role of gratitude. Subsequently, Model 5 was utilized to test the complete integrated framework, simultaneously examining empathy’s moderating effect on the direct path and the mediation process within a single conditional process model. Both applying the bootstrap method (5,000 samples) (Mallinckrodt et al., 2006). Significance was inferred from 95% CIs excluding zero (Mackinnon et al., 2004).

## 4 Results

### 4.1 Descriptive statistics and correlation analysis

Table 2 presents the kurtosis (K), skewness (S), means (M), standard deviations (SD) of the study variables, as well as the Spearman correlation results between the covariates and the main variables. The mean scores indicate that participants reported relatively high levels of perceived moral elevation, prosocial behavior, gratitude, and empathy. The mean values of both cognitive empathy and affective empathy are similar, with the mean value of cognitive empathy being relatively higher. Moral elevation (*r* = 0.508, *p* < 0.001), gratitude (*r* = 0.583, *p* < 0.001), and empathy (*r* = 0.365, *p* < 0.001) were significantly positively associated with prosocial behavior. In addition, both gratitude (*r* = 0.445, *p* < 0.001) and empathy (*r* = 0.383, *p* < 0.001) were significantly positively correlated with moral elevation.

**Table 2:** Descriptive statistics and correlation analysis of variables.

Variables	1.	2.	3.	4.	5.	6.
1. Moral elevation	1					
2. Prosocial behavior	0.508^***^	1				
3. Gratitude	0.445^***^	0.583^***^	1			
4. Empathy	0.383^***^	0.365^***^	0.449^***^	1		
5. Cognitive empathy	0.403^***^	0.379^***^	0.458^***^	0.969^***^	1	
6. Affective empathy	0.531^***^	0.428^***^	0.485^***^	0.946^***^	0.880^***^	1
*K*	−0.025	1.878	1.545	0.837	0.704	0.931
*S*	−0.423	−1.213	−1.239	0.088	−0.093	−0.030
*M*	3.859	4.020	5.587	3.580	3.362	3.617
SD	0.806	0.814	1.237	0.714	0.735	0.676

*N* = 1,261; ^***^p < 0.001.

### 4.2 Common method bias

To evaluate potential inflation of variable relationships due to shared measurement methods, Harman’s single-factor test was applied. The first factor accounted for 31.869% of the total variance, well under the 40% benchmark (Podsakoff et al., 2003), indicating that method bias was not a major concern.

### 4.3 CFA

To ensure the model fit, we conducted CFA on the measurement model of all the variables included in the study. As shown in Table 3, the model fit indices met the recommended thresholds established in previous research (Marsh and Hocevar, 1985).

**Table 3:** Model fit indices.

Fit index	Final model	Reference value
*χ*^2^/df	2.741	<3
CFI	0.936	>0.8
TLI	0.931	>0.8
RMSEA	0.037	<0.08
SRMR	0.050	<0.08
GFI	0.874	>0.8
IFI	0.936	>0.8
AGFI	0.861	>0.8

### 4.4 Mediation effect analysis

**Figure 2:**
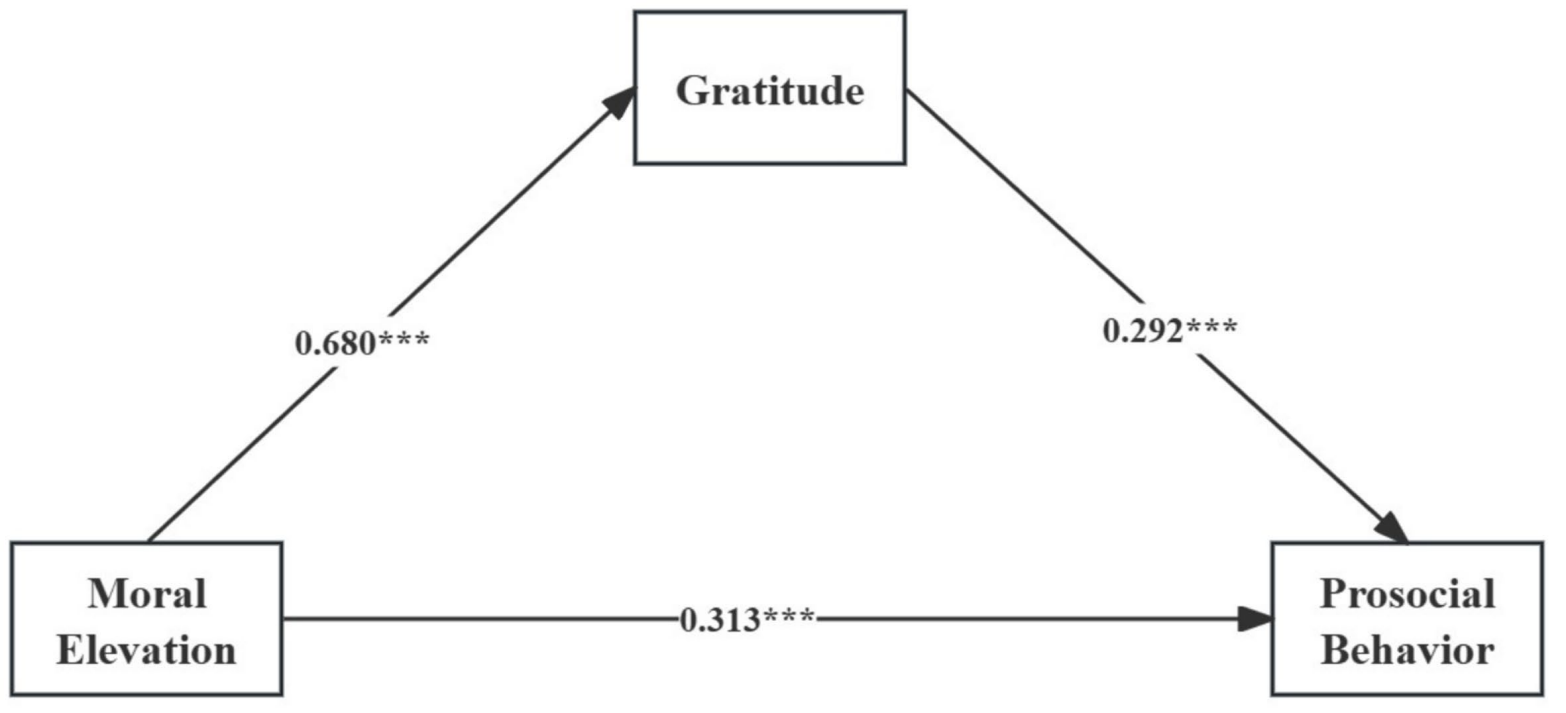
Results of mediation effects. *N* = 1,261; ^***^p < 0.001.

After establishing the prerequisites through correlation analysis, we tested a mediation model using PROCESS (Model 4) in SPSS 26.0, with 5,000 bootstrap iterations. PROCESS Model 4 tests mediation by looking at how an independent variable impacts a dependent variable through a mediator. After accounting for demographic controls (age, gender, grades, and academic discipline) the regression estimates are shown in Table 4 and Figure 2. Moral elevation significantly positively predicted gratitude (*β* = 0.680, *p* < 0.001) and prosocial behavior (*β* = 0.313, *p* < 0.001). In addition, gratitude significantly positively predicted prosocial behavior (*β* = 0.292, *p* < 0.001).

**Table 4:** Regression analysis among variables.

Outcome variable	Predictor variable	*β*	SE	*T*	Bootstrap 95% CI		*R* ^2^	*F*
					LLCI	ULCI		
Gratitude	Moral elevation	0.680^***^	0.039	17.609	0.605	0.756	0.206	65.159
	Gender	0.027	0.063	0.437	−0.095	0.150		
	Age	0.215^**^	0.065	3.307	0.087	0.342		
	Grade	−0.109^*^	0.054	−2.016	−0.216	−0.003		
	Major	−0.002	0.051	−0.045	−0.103	0.098		
Prosocial behavior	Moral elevation	0.313^***^	0.024	12.865	0.265	0.360	0.418	150.129
	Gratitude	0.292^***^	0.016	18.383	0.261	0.324		
	Gender	0.037	0.035	1.057	−0.032	0.106		
	Age	0.017	0.037	0.458	−0.055	0.089		
	Grade	0.007	0.031	0.232	−0.053	0.067		
	Major	0.026	0.029	0.900	−0.031	0.082		

*N* = 1,261; ^*^p < 0.05; ^**^p < 0.01; ^***^p < 0.001.

Furthermore, to clarify the potential confounding effects of demographic covariates, the regression coefficients and significance levels of gender, age, grade, and major were reported. The results indicated that among these covariates, age significantly predicted gratitude (*β* = 0.215, *p* < 0.01), suggesting that older students tended to experience more gratitude. In addition, grade was negatively associated with gratitude (*β* = −0.109, *p* < 0.05), indicating that students in higher grades reported slightly lower levels of gratitude. However, none of the covariates significantly predicted prosocial behavior (all *p* > 0.05), which suggests that these demographic variables did not exert substantial confounding effects on the dependent variable. These findings support the robustness of the proposed mediation model.

As shown in Table 5, the total effect of moral elevation on prosocial behavior was 0.512, and the direct effect was 0.313. When gratitude was included as a mediator, the indirect effect was 0.199, accounting for 38.867% of the total effect, indicating partial mediation. Therefore, both H1 and H2 were supported.

**Table 5:** The mediation effect of gratitude (*N* = 1,261).

Effect	Path	*β*	Percentage	Bootstrap 95% CI	
				LLCI	ULCI
Total effect	Moral elevation → Prosocial behavior	0.512	100.000%	0.463	0.560
Direct effect	Moral elevation → Prosocial behavior	0.313	61.133%	0.265	0.360
Indirect effect	Moral elevation → Gratitude → Prosocial behavior	0.199	38.867%	0.158	0.242

### 4.5 Moderation effect analysis

We tested the moderated mediation model using Model 5 of the PROCESS macro (version 4.2) in SPSS. PROCESS Model 5 is designed to examine both mediation and moderation simultaneously, making it the ideal choice for our analysis. By presenting all results from Model 5, we are able to comprehensively assess how empathy moderates the relationship between gratitude (the mediator) and prosocial behavior (the outcome), after confirming the mediating effect of gratitude established in Model 4. Empathy represents the mean of both affective empathy and cognitive empathy. The analysis results are presented in Table 6.

**Table 6:** The moderation effect of empathy (*N* = 1,261).

Outcome variable	Predictor variable	*β*	SE	*T*	Bootstrap 95% CI		*R^2^*	*F*
					LLCI	ULCI		
W = Empathy								
Gratitude	Moral elevation	0.680^***^	0.039	17.609	0.605	0.756	0.206	65.159
Prosocial behavior	Moral elevation	0.285^***^	0.025	11.456	0.236	0.333	0.432	119.131
	Gratitude	0.257^***^	0.017	14.948	0.224	0.291		
	Empathy	0.076^**^	0.028	2.727	0.021	0.131		
	Moral elevation **×** Empathy	−0.115^***^	0.023	−4.999	−0.160	−0.070		
W = Cognitive empathy								
Gratitude	Moral elevation	0.680^***^	0.039	17.609	0.605	0.756	0.206	65.158
Prosocial behavior	Moral elevation	0.283^***^	0.025	11.340	0.234	0.332	0.432	118.943
	Gratitude	0.257^***^	0.017	14.956	0.224	0.291		
	Cognitive empathy	0.077^**^	0.027	2.809	0.023	0.131		
	Moral elevation **×** Cognitive empathy	−0.106^***^	0.022	−4.788	−0.150	−0.063		
W = Affective empathy								
Gratitude	Moral elevation	0.680^***^	0.039	17.609	0.605	0.756	0.206	65.158
Prosocial behavior	Moral elevation	0.262^***^	0.026	9.959	0.210	0.314	0.439	122.273
	Gratitude	0.250^***^	0.017	14.584	0.216	0.284		
	Affective empathy	0.097^**^	0.032	3.024	0.034	0.159		
	Moral elevation **×** Affective empathy	0.148^***^	0.024	−6.159	−0.195	−0.101		

^**^p < 0.01; ^***^p < 0.001.

As shown in Table 6, both dimensions of empathy significantly moderated the relationship between moral elevation and prosocial behavior. Specifically, the interaction between moral elevation and prosocial behavior demonstrated a negative moderating effect (*β* = −0.115, *p* < 0.001), indicating that as the level of empathy increased, the facilitative effect of moral elevation on prosocial behavior weakened. Moreover, cognitive empathy played a significant role in this moderating effect (*β* = −0.106, *p* < 0.001). Notably, affective empathy also exhibited a strong negative moderating effect, with an even more pronounced negative moderating effect (*β* = −0.148, *p* < 0.001). When the moderating effect of empathy was added to the model, the indirect effect of moral elevation on prosocial behavior through gratitude decreased, highlighting the important role of empathy in moderating the indirect pathway. The direct effect of moral elevation on prosocial behavior also showed a slight reduction after considering empathy as a moderator, indicating that empathy not only affects the strength of the mediation but also alters the direct relationship between moral elevation and prosocial behavior. These results suggest that both dimensions of empathy play an important moderating role in the impact of moral elevation on prosocial behavior.

**Figure 3:**
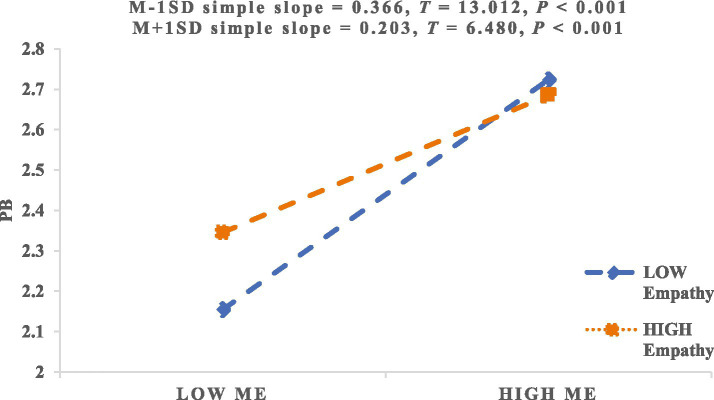
The moderating effect of empathy on the relationship between moral elevation and prosocial behavior.

To further illustrate this effect, we plotted simple slope lines for the relationship between moral elevation and prosocial behavior at low and high levels of empathy (see Figure 3). The results indicated that for college students with low levels of empathy, moral elevation significantly predicted prosocial behavior (*β*_simple_ = 0.366, *p* < 0.001). However, among those with high empathy levels, the positive association between the two variables was weaker (*β*_simple_ = 0.203, *p* < 0.001). These findings suggest that as empathy increases, the predictive effect of moral elevation on prosocial behavior gradually decreases. Therefore, H3a, H3b and H3c were supported.

**Figure 4:**
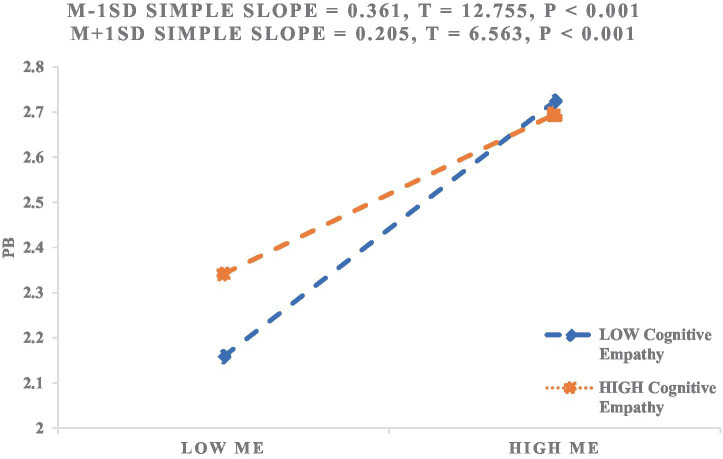
The moderating effect of cognitive empathy on the relationship between moral elevation and prosocial behavior.

**Figure 5:**
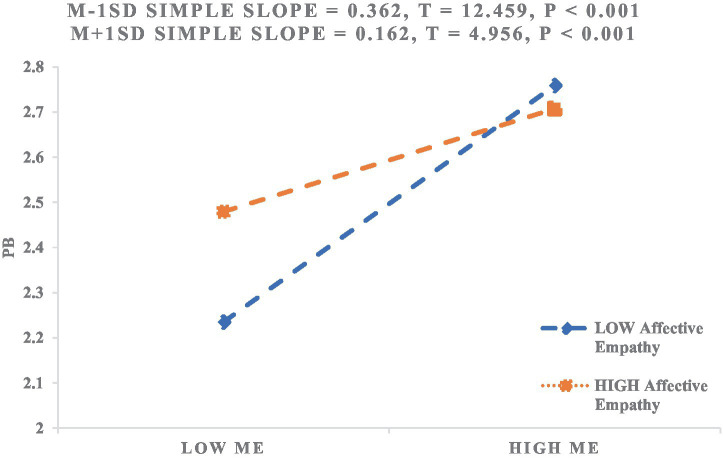
The moderating effect of affective empathy on the relationship between moral elevation and prosocial behavior.

Additionally, we plotted the simple slope relationships between moral elevation and prosocial behavior under the two dimensions of cognitive empathy and affective empathy, as shown in Figures 4 and 5. Figure 4 indicates that for college students with low levels of cognitive empathy, moral elevation significantly predicts prosocial behavior (*β*_simple_ = 0.361, *p* < 0.001). However, for students with high levels of cognitive empathy, the positive correlation between moral elevation and prosocial behavior weakens (*β*_simple_ = 0.205, *p* < 0.001). The moderating effect of cognitive empathy is similar to the overall empathy moderating effect.

As shown in Figure 5, for students with low levels of affective empathy, moral elevation significantly predicts prosocial behavior (*β*_simple_ = 0.362, *p* < 0.001). However, for students with high levels of affective empathy, the positive correlation between moral elevation and prosocial behavior weakens (*β*_simple_ = 0.162, *p* < 0.001). Although the moderating mechanism is similar to that of empathy and cognitive empathy, for students with high levels of affective empathy, the impact of moral elevation on prosocial behavior is relatively weakened.

## 5 Discussion

Grounded in Social Intuitionist Model (Haidt, 2001) and the Empathy–Altruism Hypothesis (Batson, 1991), this study examined factors related to prosocial behavior among college students. Cross-sectional analysis demonstrated that moral elevation promotes prosociality both directly and indirectly through the partial mediation of gratitude. Moreover, empathy negatively moderated the direct link between these variables. The following discussion elaborates on these results within established theoretical frameworks.

First, the study confirmed a significant positive association between moral elevation and prosocial behavior among college students, which is consistent with findings reported by Ding et al. (2018). According to the Social Intuitionist Model (Haidt, 2001), moral elevation, as a form of positive moral emotion, may serve as a psychological bridge linking moral cognition and moral behavior, thereby fostering individuals’ prosocial tendencies. Specifically, moral emotions arouse attention to others’ welfare and social norms, which can be associated with intrinsic altruistic motivation and a sense of social responsibility (Haidt, 2003b). Positive emotions function as both immediate facilitators of helpful action and long-term enablers of prosocial habit formation by shaping affective and motivational systems. From the perspective of emotional arousal mechanisms, moral elevation is thought to activate individuals’ moral cognitive systems and evoke beliefs in the inherent goodness of human nature (Schnall et al., 2010). In this process, individuals do not merely admire others’ virtuous acts but are also motivated by a desire to become “that kind of person” themselves. Such motivational alignment facilitates the internalization of moral norms and promotes consistent prosocial action. Moreover, the Social Intuitionist Model emphasizes that moral emotions function not only as affective responses but also as regulators of social behavior. Moral elevation, by evoking feelings such as admiration and inspiration, enhances individuals’ identification with social norms, moral values, and group belongingness (Freeman et al., 2009), which may in turn relate to behaviors that support, assist, and cooperate with others in ways that facilitate social cohesion. Taken together, moral elevation is not merely an emotional reaction to witnessing virtuous conduct, but a socially embedded psychological experience that has the potential to motivate morally congruent action. By eliciting positive emotions, reinforcing moral identification, and expanding motivational readiness, moral elevation may be associated with more frequent and sustained engagement in prosocial behavior.

The second major finding revealed a significant partial mediating role of gratitude in the association between moral elevation and prosocial behavior. Moral elevation, experienced in response to virtuous actions observed in others, may be linked to increased gratitude, which in turn may contribute to greater prosocial engagement. The indirect pathway accounted for 38.867% of the total effect (*β* = 0.199), indicating partial mediation. Consistent with prior evidence, this result aligns with findings reported by Sheng and Fang (2024) and Zhu et al. (2024). According to Social Intuitionist Model (Haidt, 2001), moral emotions such as gratitude and elevation are conceptualized as bridges between moral cognition and moral behavior. When individuals witness virtuous acts performed by others, the positive emotional responses associated with moral elevation may give rise to socially oriented emotions such as gratitude (Algoe and Haidt, 2009). Gratitude not only enhances awareness of others’ kindness but also may activate internal motivations oriented toward reciprocity and voluntary prosocial action (McCullough et al., 2001). Moreover, from the perspective of socially functional emotions, gratitude is considered to contribute to the maintenance of interpersonal connections and social cohesion (Algoe, 2012). It may also function as a moral motive that transforms individuals’ emotional experiences of moral elevation into actual helping behaviors (Wong et al., 2024). Therefore, moral elevation can provide the emotional foundation for gratitude, and gratitude may serve as a mechanism that translates abstract moral motivation into concrete behavioral tendencies, reinforced through enhanced self-identification. Regarding the role of gratitude in this association, we clarify that gratitude is not simply a mediator but functions as a key emotional mechanism that helps individuals translate their moral emotions into prosocial actions. Gratitude provides the necessary emotional drive to move from abstract moral thoughts to actual behavior. This process aligns with the idea that emotions such as gratitude enable individuals to act on their moral values, transforming them into social actions.

Finally, the findings demonstrated a significant negative moderating effect of empathy on the link between moral elevation and prosocial behavior, indicating that the positive association between the two variables may weaken as empathy levels increased. This result is consistent with the findings of Li et al. (2022). The Empathy–Altruism Hypothesis (Batson, 1991) posits that empathy leads individuals to engage in prosocial behavior based on intrinsic altruism, not external rewards. Highly empathetic individuals are often rich in prosocial resources, gained through repeated emotional experiences. As a result, they may not rely on the additional broadening effects of moral elevation to engage in prosocial behavior (Morelli et al., 2015). Compared to their high-empathy counterparts, those with limited empathic capacity often exhibit reduced emotional resources and less internalized motivation for prosocial engagement in routine interactions. Consequently, they may be more susceptible to the broadening effects of external positive emotions (Fu et al., 2022). This suggests that moral elevation, as an emotional moral motivator, may be associated with prosocial behavior depending on an individual’s baseline level of empathic resources. When empathy is low, moral elevation may provide an additional emotional drive that could enhances prosocial behavior. However, when empathy is high, prosocial behavior is likely sustained by internal motivation, and the incremental effect of moral elevation becomes relatively limited (Van de Vyver and Abrams, 2017). In summary, empathy helps determine the strength of the connection between moral elevation and prosocial behavior by influencing the psychological resources individuals bring to the situation. For individuals with high empathy, abundant internal resources may reduce the marginal effect of moral elevation. Conversely, for individuals with low empathy, moral elevation could serve as a more critical catalyst for activating prosocial behavior given their comparatively limited internal resources.

Notably, the moderating roles of cognitive and affective empathy in this association appear to diverge. Specifically, affective empathy seems to have a stronger moderating effect, while the effect of cognitive empathy appears to be more moderate. Affective empathy involves an individual’s emotional resonance with others’ feelings, and this emotional resonance can drive prosocial behavior. When individuals experience others’ emotions, their internal emotional motives may prompt them to take action (Batson, 1991). Individuals with high levels of affective empathy typically maintain prosocial behavior through abundant emotional resources, meaning that external emotional incentives (such as moral elevation) may have a weaker facilitative effect on them (Morelli et al., 2015). Our data show that the moderating effect of affective empathy is negative (*β* = −0.148, *p* < 0.001), suggesting that as the level of affective empathy increases, the facilitative effect of moral elevation on prosocial behavior weakens. This finding can be further explained from the perspective of emotional regulation and resource depletion theory. High affective empathy enables individuals to build emotional resources over time, allowing them to sustain prosocial behavior without relying on external emotional cues (Gross and John, 2003). However, an excess of emotional resources could lead to emotional “overload” or emotional regulation “saturation,” thereby weakening the influence of external emotional stimuli (such as moral elevation) on prosocial behavior. Cognitive empathy emphasizes the rational appraisal of others’ emotional states rather than direct emotional resonance (Powell and Roberts, 2017; Thompson et al., 2022). Consequently, within the moral elevation context, the prosocial behavior of individuals high in cognitive empathy is driven primarily by analytical judgment, resulting in a more attenuated moderating effect (*β* = −0.106, *p* < 0.001). Unlike affective empathy, which leverages emotional contagion to bypass or amplify motives, cognitive empathy relies on deliberate evaluation, making it less sensitive to the emotional incentives of elevation. This divergence underscores their distinct mechanisms: affective empathy promotes prosociality through affective mirroring, whereas cognitive empathy operates through cognitive understanding and normative assessment.

## 6 Implications

Focusing on undergraduate populations, the present research systematically explores the psychological pathways through which moral elevation may influence prosocial behavior. The findings provide insights into moral-emotional functioning in emerging adults and offer actionable insights for institutional practices related to character development, emotional health promotion, and student well-being services.

### 6.1 Theoretical implications

First, it advances the understanding of how positive moral emotions, specifically moral elevation, can influence prosocial behavior. By grounding the analysis in Social Intuitionist Model (Haidt, 2001), the study clarifies the psychological mechanisms through which moral emotions may act as a bridge between moral cognition and moral behavior. Although previous research has shown that moral elevation can elicit prosocial tendencies (Algoe and Haidt, 2009; Thomson and Siegel, 2017), the current findings suggest that this effect may not be equally strong across individuals. Empathy, as a relatively stable moral-emotional trait, may moderate the effect of moral elevation on prosocial behavior under certain conditions. These finding highlights that the social function of positive emotions may be influenced by individual differences and should not be assumed to operate uniformly across populations.

Second, this study deepens the theoretical understanding of empathy’s role in moral behavior by illustrating that its effects may be context-dependent and nonlinear. While empathy has traditionally been viewed as universally beneficial for moral evaluation and prosocial behavior (Jolliffe and Farrington, 2006; Lockwood et al., 2014), the present study suggests that excessive empathy, particularly in morally sensitive individuals, could lead to emotional strain or burnout, thereby impairing behavioral engagement (Huang et al., 2025). This insight advances the theoretical literature by proposing that the impact of empathy on behavior is not always positive and may be constrained by emotional resources, especially in emotionally intense moral situations. Thus, the study highlights the potential costs of empathy, offering a more balanced view of its role in moral and prosocial development.

Finally, this study constructs a mediated pathway from moral elevation to prosocial behavior via gratitude, and further introduced empathy as a boundary condition. The resulting moderated mediation model offers an integrated framework for understanding how internal emotional experiences may be translate into observable social behaviors. This research contributes to bridging moral and socio-emotional domains and opens new avenues for applying positive psychological principles in educational settings and personality formation.

### 6.2 Practical implications

First, the study supports the notion that moral elevation may be linked to prosocial tendencies, indicating that activating positive moral emotions could influence individuals’ inclination to engage in prosocial behavior. This finding has relevance for the development of moral education programs in universities, as it highlights the importance of not only imparting behavioral norms but also fostering emotional engagement. Educators may guide students to become more attuned to others’ well-being and enhance their moral sensitivity through activities such as sharing examples of virtuous actions, facilitating moral reflection, and organizing service-learning projects. These activities should focus on cultivating emotional connections to ethical values, as they appear to be critical for motivating prosocial actions.

Secondly, the study provides insights into gratitude as a mediator, offering a practical basis for initiatives aimed at promoting gratitude and enhancing cooperation within university communities. Universities could consider implementing programs that increase gratitude awareness and encourage emotional expression—such as gratitude journaling, peer-sharing sessions, or gratitude-themed events. These initiatives may help students express appreciation and develop concern for others, thus strengthening interpersonal bonds and fostering a sense of social responsibility.

Finally, the finding that empathy negatively moderates the relationship between moral elevation and prosocial behavior introduces a unique implication. Specifically, it suggests that excessive empathy may lead to emotional strain, potentially reducing individuals’ engagement in prosocial actions. This insight emphasizes the need for developing emotional regulation skills in university settings. Educators are encouraged to incorporate interventions, such as mindfulness training, self-compassion programs, and resilience-building workshops, to help students manage their empathic responses. These interventions could assist students in maintaining healthy emotional boundaries, ensuring that moral motivation can be translated into prosocial behavior effectively.

## 7 Limitations and future directions

One limitation of this study is the use of a cross-sectional design, which limits the ability to infer causal relationships among the study variables. Given the potentially evolving nature of moral elevation, gratitude, and prosocial behavior, longitudinal or intervention-based research is needed to examine how these constructs interact over time and under varying conditions. For example, Immordino-Yang et al. (2009), using an fMRI experiment, found that emotional responses of admiration and compassion—elicited by real-life stories—activated brain regions associated with social cognition, empathy, and reward processing, such as the anterior cingulate cortex and nucleus accumbens. These neural activations were significantly related to altruistic behavior, suggesting that moral elevation has the potential to engage the neurobiological foundations of prosociality.

Another limitation is the reliance on self-reported measures, which may introduce biases such as overstating moral self-image or misrepresenting emotional experiences, potentially affecting the validity of results concerning ethical constructs. To improve objectivity and ecological validity, future research could integrate multiple data sources, including peer evaluations, teacher assessments, and behavioral measures such as observed helping tasks.

The sample was drawn from universities in mainland China, which provides some degree of representativeness, though there may be regional limitations in terms of cultural background and social values. Cultural contexts often shape how individuals experience moral emotions and engage in prosocial behaviors. Culturally specific factors such as Zhongyong thinking and collectivism may shape the observed patterns (Xiao et al., 2021; Zhou, 2025). For instance, Zhou (2025) found that traditional Chinese values rooted in Zhongyong philosophy directly influence college students’ prosocial behavior. Therefore, future research should examine the proposed model in diverse cultural contexts to assess its generalizability and cultural adaptability.

Finally, while data were collected from multiple universities across diverse regions and institutional types, we acknowledge that the sample may not fully capture the complexity of China’s higher education landscape. Future studies could explore stratified sampling across broader provinces and include additional university categories (e.g., vocational colleges, elite institutions) to further enhance generalizability.

## 8 Conclusion

Prosocial behavior is essential for cultivating college students’ moral awareness and sense of social responsibility. Yet, most existing studies have concentrated on children and adolescents, leaving limited insight of this phenomenon in the college population. Grounded in the Social Intuitionist Model and the Empathy–Altruism Hypothesis, the present study explored the effect of moral elevation on prosocial behavior, with gratitude as a mediator and empathy as a moderator. The survey results suggested a significant positive association between moral elevation and prosocial behavior. Gratitude acted as a mediator in this relationship, accounting for 38.867% of the total effect. Moreover, empathy negatively moderated the direct path from moral elevation to prosocial behavior, indicating that the positive association between the two tended to weaken as empathy levels increased. Our work expands moral psychological frameworks through a clearer mapping of prosocial pathways, thereby informing interventions in educational, organizational, and social governance contexts. The cross-sectional nature of this study precludes causal inferences, and reliance on self-reported data may introduce social desirability or response biases. Future research should employ longitudinal designs or experimental manipulations to establish causality and further refine the current model.

## Data Availability

The datasets presented in this study can be found in online repositories. The names of the repository/repositories and accession number(s) can be found in the article/supplementary material.
